# Assessment of arterial stiffness using pulse wave velocity in tacrolimus users the first year post kidney transplantation: a prospective cohort study

**DOI:** 10.1186/s12882-015-0092-7

**Published:** 2015-07-02

**Authors:** Kelly Ann Birdwell, Gilad Jaffe, Aihua Bian, Pingsheng Wu, Talat Alp Ikizler

**Affiliations:** Division of Nephrology and Hypertension, Vanderbilt University Medical Center, 1161 21st Avenue, S-3223 MCN, Nashville, TN 37232 USA; University at Buffalo School of Medicine and Biomedical Sciences, 3435 Main Street, Buffalo, NY 14260 USA; Department of Biostatistics, Vanderbilt University Medical Center, 2525 West End Avenue, Suite 11000, Nashville, TN 37203 USA

**Keywords:** Transplant, Cardiovascular, Outcome, Diabetes, Arterial stiffness

## Abstract

**Background:**

The leading cause of death in end stage renal disease is cardiovascular disease (CVD). Kidney transplantation is associated with improved survival over dialysis. We hypothesized that arterial stiffness, a marker of CVD, would improve in patients post kidney transplant, potentially explaining one mechanism of survival benefit from transplant.

**Methods:**

After obtaining Institutional Review Board approval and informed consent, we performed a longitudinal prospective cohort study of 66 newly transplanted adult kidney transplant recipients, using aortic pulse wave velocity (PWV) to assess arterial stiffness over a 12 month period. All patients were assessed within one month of transplant (baseline) and 12 months post transplant. The primary outcome was change in PWV score at 12 months which we assessed using Wilcoxon Signed Rank test. Secondary analyses included correlation of predictors with PWV score at both time points.

**Results:**

The median age of the cohort was 49.7 years at transplant, with 27 % Black and 27 % female. At baseline, 43 % had tobacco use, 30 % had a history of CVD, and 42 % had diabetes. Median baseline calcium was 9.1 mg/dL and median phosphorus was 5.1 mg/dL. Median PWV score was 9.25 and 8.97 m/s at baseline versus month 12, respectively, showing no significant change (median change of −0.07, p = 0.7). In multivariable regression, subjects with increased age at transplant (p = 0.008), diabetes (p = 0.002), and a higher baseline PWV score (p < 0.001) were at increased risk of having a high PWV score 12 months post transplant.

**Conclusion:**

Aortic arterial stiffness does not progress in the first year post kidney transplant. Increasing age, diabetes, and higher baseline PWV score identify patients at risk for increased arterial stiffness. Further research that assesses patients for greater than one year and includes a control dialysis group would be helpful in further understanding the change in arterial stiffness post transplantation.

## Background

Cardiovascular disease (CVD) exerts a heavy burden in patients with chronic kidney disease (CKD), and it is the leading cause of death in the end stage renal disease (ESRD) population [1–3]. Contributors to CVD in ESRD include vascular arterial calcifications from abnormal mineral metabolism and inflammation related to poor kidney function. Kidney transplantation leads to improved survival compared to wait-listed dialysis patients, making it the renal replacement therapy of choice in eligible patients [4]. The reasons for this survival advantage are likely multifactorial, but include improved cardiovascular risk as a result of return of kidney function [5–7]. Even so, kidney transplant recipients continue to have a high burden of CVD, with CVD being the number one cause of death with a functioning transplant [1].

Increased aortic arterial stiffness is strongly associated with the development of CVD and is an independent predictor of cardiac and all-cause mortality [8]. Aortic pulse wave velocity (PWV) provides a non-invasive method to assess aortic arterial stiffness [8, 9]. Though often used in the research setting, several studies support using PWV in the clinic based on its reported associations with cardiovascular risk and all-cause mortality in a variety of patient populations, with one meta-analysis showing each increase of 1 m/s in aortic PWV being associated with a 15 % risk-adjusted increase in both cardiovascular and all-cause mortality [10]. Similar findings have been demonstrated specifically in patients with CKD and on maintenance dialysis, with PWV increasing over time in maintenance dialysis patients [11–14]. Higher aortic PWV has also been associated with all-cause mortality and cardiovascular events in few studies of prevalent kidney transplant recipients, but less is known about changes that may occur in arterial stiffness over time after successful kidney transplantation [15, 16].

We hypothesized that aortic arterial stiffness would be improved in patients receiving a kidney transplant, potentially explaining one mechanism of survival benefit from transplant. In this study we examined a prospective cohort of 66 kidney transplant recipients with aortic PWV measurements and assessed the changes in aortic arterial stiffness over the first year post kidney transplant.

## Methods

### Study design

All study procedures were approved by the Institutional Review Board (IRB) of Vanderbilt University Medical Center (VUMC). Informed consent was obtained from all study participants. We conducted a single-center, prospective cohort study of 66 newly transplanted adult kidney transplant recipients, using pulse wave velocity (PWV) to assess arterial stiffness over a 12 month period. Kidney transplant recipients were consecutively recruited from the VUMC Renal Transplant Clinic from August 2009 through September 2012. Inclusion criteria included patients aged ≥ 18 who were undergoing or had recently undergone kidney transplantation. Participants were excluded if they were unwilling to participate or had atrial fibrillation, since this condition makes pulse wave velocity testing uninterpretable. Induction therapy was alemtuzumab with methylprednisolone in 85 % of patients. All patients were maintained on tacrolimus and mycophenolate. Maintenance steroids were used in 9 %. Tacrolimus was dosed to a 12 h trough blood concentration of 8–10 ng/ml the first 6 months, then of 6–8 mg/ml for months 6 through 12.

The study was composed of two visits, with patients assessed within 1 month of transplant (baseline) and 12 months post transplant. Demographic, medical, and social data was collected from each participant and confirmed in the electronic medical record. Participants underwent pulse wave velocity testing by a trained professional at the Vanderbilt Clinical Research Center using the SphygmoCor CPV Pulse Wave Velocity system (AtCor Medical, Sydney, Australia). Briefly stated, pulse wave velocity is the time it takes the arterial pressure wave to travel between two arterial sites, where PWV = distance (meters)/transit time (seconds). A higher PWV positively correlates with increased arterial stiffness. A more detailed description of pulse wave velocity procedure has been previously published [8]. In this study the carotid to femoral distance was selected, representing aortic arterial stiffness. The distance was measured by subtracting the distance between the carotid site and sternal notch from the distance between the sternal notch and the femoral site. For participants who were hemodialysis patients with vascular accesses, measures were taken on the non-access side. After resting 10 min in a supine position, hemodynamic measurements were obtained for each participant. All measurements were performed twice with the average of the two values used for analysis.

### Statistical analysis

We expressed descriptive statistics as proportions for categorical variables and as means and standard deviations, or medians and interquartile ranges for continuous variables, depending on the distribution of the variables. The primary outcome was change in PWV score at 12 months from baseline, which we assessed using Wilcoxon Signed Rank test. We further assessed the change in PWV score stratified by subjects’ diabetes status at baseline. Secondary analyses included predictors of PWV score at baseline and at 12 months post transplant. Spearman’s rank correlation was used to test the association of age at baseline with PWV score at baseline and 12 months as well as the association of PWV score at baseline and at 12 months. We further conducted multivariable regression to determine predictors for both baseline and 12 month PWV measures. Age at transplantation, race, gender, diabetes status, and time in months on dialysis at baseline were included as potential predictors. Baseline PWV score was also included in the multivariable model to predict PWV score at 12 month post transplant. Normality of residuals of all linear models was diagnosed figuratively. We conducted all analyses using R-software version 2 · 11 · 1 (www.r-project.org) and used a two-sided 5 % significance level for all statistical analyses.

## Results

Seventy-eight kidney transplant recipients were enrolled into the study. Sixty-six of these had pulse wave velocity measures available at baseline and at 12 months post transplant. Characteristics of the study cohort are presented in Table 1. The median age of the cohort was 49.7 years at transplant, with 27 % black and 27 % female. At baseline, 43 % had tobacco use, 27 % had a history of CVD, and 42 % had diabetes. Mean blood pressure was 136/79 mmHg at baseline and 131/77 mmHg at month 12, with 94 % of the cohort with pre-existing diagnosis of hypertension. Baseline calcium was 9.1 mg/dL and phosphorus was 5.1 mg/dL. The median duration of dialysis prior to transplant was 1.74 years, though 18 % of recipients had no dialysis time (pre-emptive transplant). At baseline, median serum creatinine was 8.4 mg/dL and estimated glomerular filtration rate (eGFR) was 10 mL/min/1.73 m^2^. Median serum creatinine 12 months post kidney transplant was 1.4 mg/dL, corresponding to an eGFR 61 mL/min/1.73 m^2^.

**Table 1:** Characteristics of the 66 kidney transplant recipients

Characteristic	
Age at transplant (years)	49.7 (41, 61)
Race	
White	46 (70 %)
Black	18 (27 %)
Other	2 (3 %)
Sex (female)	18 (27 %)
Tobacco use	
Never	37 (57 %)
Current	5 (8 %)
Former	23 (35 %)
Cardiovascular disease pre transplant^a^	18 (27 %)
Diabetes pre transplant	28 (42 %)
Primary cause end stage renal disease	
Diabetes	21 (32 %)
Hypertension	13 (20 %)
Glomerulonephritis	11 (17 %)
Cystic	10 (15 %)
Other	11 (17 %)
Serum creatinine (mg/dL)	
Pre transplant	8.4 (6.2, 9.7)
1 month post transplant	1.5 (1.2, 1.7)
12 months post transplant	1.4 (1.2, 1.6)
Estimated glomerular filtration rate (mL/min/1.73 m^2^)	
Pre transplant	8.3 (6, 10.5)
1 month post transplant	53.7 (45.5, 69.9)
2 months post transplant	61 (47.8, 70.4)
Calcium pre transplant (mg/dL)	9.1 (8.5,9.5)
Phosphorus pre transplant (mg/dL)	5.1 (4.2, 6.5)
Systolic blood pressure (mm Hg)	
Baseline	136.2 (123, 149)
Month 12	131.3 (120.5, 143)
Diastolic blood pressure (mm Hg)	
Baseline	79 (72, 84)
Month 12	77.1 (42, 83)
Time on dialysis prior to transplant (days)	636 (362, 1254)

Values expressed as median (25th, 75th percentiles) or number (percent)

^a^Cardiovascular disease includes coronary artery disease, stroke, and peripheral vascular disease

**Fig. 1:**
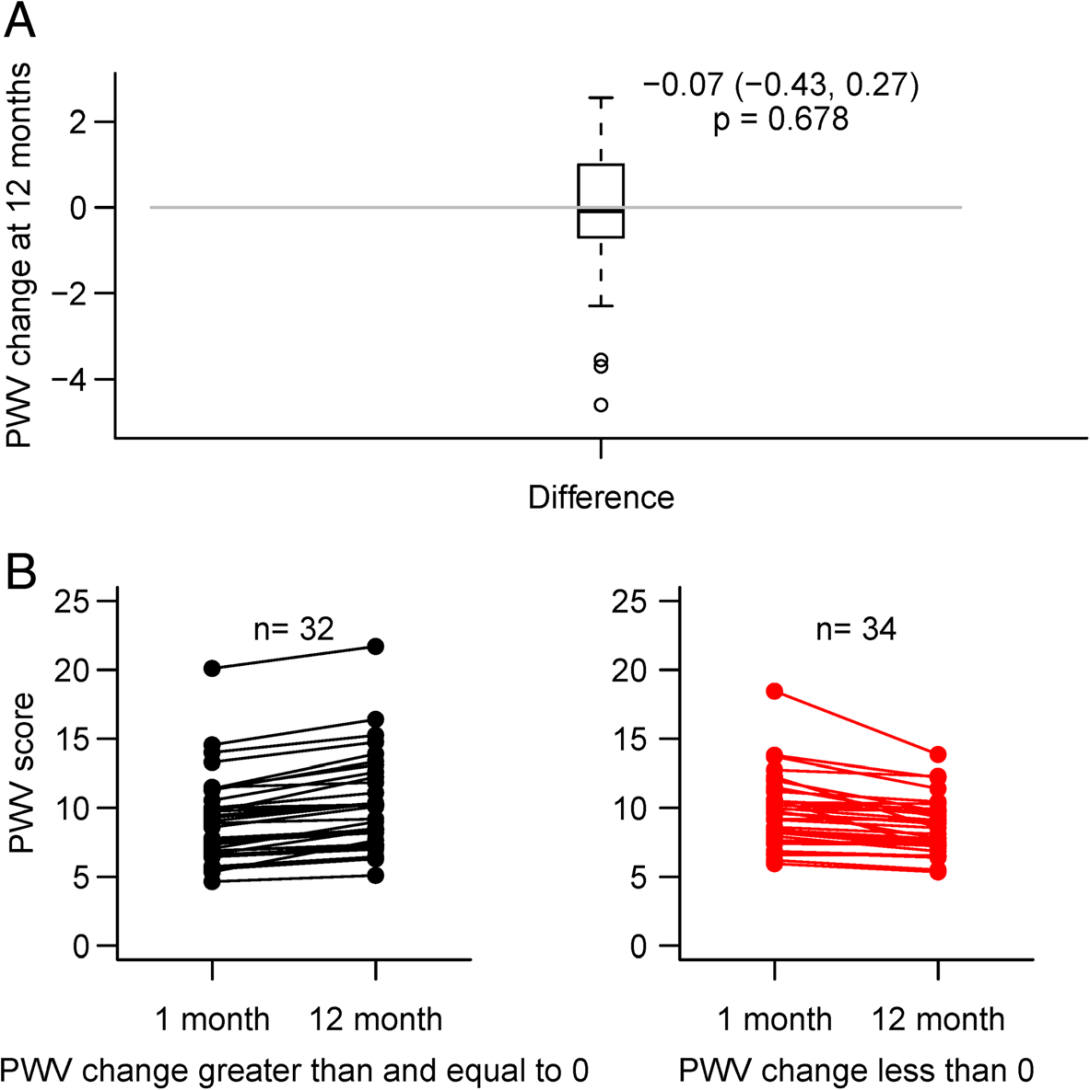
**a** Box plot of change in PWV from baseline to month 12 in 66 kidney transplant recipients. **b** Individual line plots of PWV change from baseline to month 12 in each kidney transplant recipient, stratified by positive or negative change

**Fig. 2:**
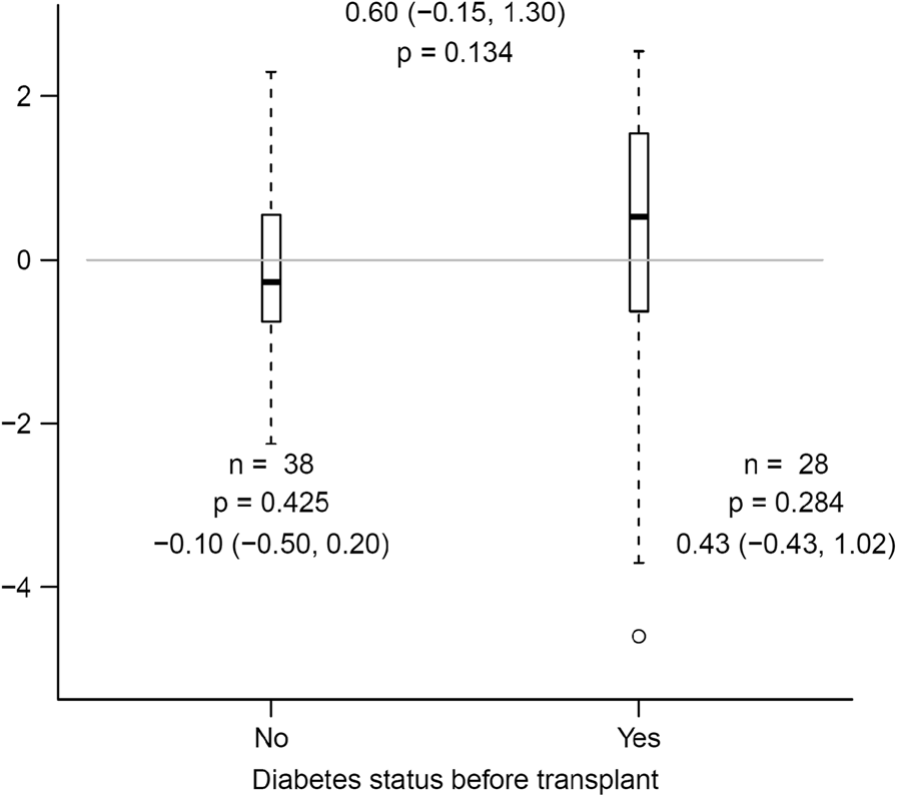
Box plot of change in PWV from baseline to month 12 in kidney transplant recipients with and without diabetes prior to transplant

The median PWV was 9.25 m/s at baseline. At month 12 it was 8.97 m/s, showing no significant change in PWV over the study period (median change of −0.07, p = 0.7; Fig. 1a). Twenty-five (37.9 %) of the participants had a PWV at baseline greater than or equal to 10 m/s, indicating severe arterial stiffness at the time of transplant. Overall, about half the kidney transplant recipients showed an increase in PWV over 12 months, while the other half decreased, with no relation to PWV at baseline (Fig. 1b). Prior to analysis, we hypothesized diabetes would have an effect on PWV. Therefore we assessed the change in PWV in kidney transplant recipients with and without diabetes. As seen in Fig. 2, though patients without diabetes trended to a decrease in PWV and patients with diabetes an increase in PWV, these changes were not significant within or between the groups. Overall 8 new cardiovascular events were observed during the 12 month follow up period.

Our secondary analyses centered on predictors of PWV at baseline and at 12 months. Using Spearman correlation, age strongly correlated with pulse wave velocity at baseline, showing increasing age with increasing baseline value (rho = 0.59, P < .001). Neither time on dialysis nor the calcium-phosphorus product prior to transplant correlated with PWV at baseline. For PWV at 12 months, both PWV at baseline (rho = 0.87, p < .001) and age (rho = 0.61, P < .001) were significantly correlated. No significant correlation was seen between serum creatinine at 12 months and PWV at 12 months. Results for multivariable regression are shown in Table 2. Increased age at transplant and presence of diabetes were significant predictors of higher PWV at baseline. Similarly, significant predictors of higher PWV at 12 months were increased age and diabetes at time of transplant, as well as higher PWV at baseline.

**Table 2:** Multivariable linear regression model of PWV in kidney transplant recipients

Covariate	Effect	S.E.	95 % CI	P
Predictors of PWV at baseline				
Age at transplant	2.8	0.5	1.8–3.8	<0.0001
Non-white race	0.4	0.6	−0.8–1.6	0.5
Female sex	0.5	0.6	−0.7–1.7	0.4
Diabetes pre transplant	2.5	0.6	1.4–3.6	<0.0001
CVD pre transplant^a^	0.9	0.6	−0.4–2.1	0.2
Time on dialysis	0.2	0.3	−0.3–0.7	0.5
Predictors of PWV at 12 months				
PWV at baseline	2	0.3	1.5–2.6	<0.0001
Age at transplant	1.1	0.4	0.3–1.9	0.01
Non-white race	0.4	0.4	−0.4–1.2	0.4
Female sex	0.5	0.4	−0.3–1.3	0.2
Diabetes pre transplant	1.5	0.4	0.6–2.3	0.001
CVD pre transplant^a^	0.04	0.4	−0.8–0.9	0.9
Time on dialysis	0.2	0.2	−0.2–0.5	0.4

^a^Cardiovascular disease (CVD) includes coronary artery disease, stroke, and peripheral vascular disease

## Discussion

Patients who undergo kidney transplantation have improved survival and lower cardiovascular risk relative to wait-listed dialysis patients. This suggests that transplant may positively impact cardiovascular risk. Though this effect likely occurs through several processes, here we examined the effect of kidney transplantation on arterial stiffness by assessing PWV. In this prospective study of 66 new kidney transplant recipients, we showed that there was no change in aortic PWV over the first year post kidney transplant, suggesting arterial stiffness does not progress during this time period. Increasing age, having diabetes and higher baseline PWV score were associated with increased PWV score at 12 months and may help identify patients with increased arterial stiffness.

Previous studies have suggested that PWV progresses with time on dialysis. For example, Utescu and colleagues showed an annual increase of 0.84 m/s per year (95 % CI 0.50−1.12 m/s per year) in a cohort of 109 hemodialysis patients [12]. In another study of 88 wait-listed patients in which 39 were transplanted and 49 were not, during one year follow up no difference was seen in PWV between the 2 groups, though follow up time for those transplanted was short (median 6.3 months) [17]. In our study, in contrast to dialysis patients, we did not see PWV progression over the first year post transplant. One interpretation could be that restoration of kidney function with transplant has a positive impact on slowing the process of arterial stiffness. However, further study is needed to see if this trend continues over longer time periods, and more importantly if this is associated with decreased CVD events. Given the adverse metabolic effects of routinely used immunosuppression drugs, it is possible that with more time the balance shifts the opposite direction.

For kidney transplant recipients, prior cross-sectional studies have shown that transplant recipients have higher aortic PWV measurements than historical healthy controls. The reported median normal values for a large European population of individuals aged 40–49 was 6.9 m/s and aged 50–59 was 8.1 m/s compared to 9.25 m/s in our patient cohort with a median age of 49.7 years [9]. In a retrospective study, 106 patients in Lebanon at a mean 54.1 months post transplant had mean aortic PWV of 11.1 ± 2.8 m/s compared to 8.7 ± 1.6 m/s in a previously studied healthy male cohort. The mean PWV value in that study was much higher than what we observed in our cohort. In addition, the transplant recipients who had experienced more cardiovascular events at time of PWV assessment had a significantly higher aortic PWV than those who did not (12.8 ± 4.3 vs 10.9 ± 2.5 m/s, respectively, p < 0.05) [18]. Similarly a study from Belgium of primarily white patients showed a higher baseline measure of PWV was associated with more cardiovascular events after a mean follow up of 5 years (11.2 vs 9.2 m/s, p < 0.001) [19].

These prior studies had single time points of PWV assessments of prevalent transplant recipients, with patients enrolled at varying times post transplantation. Our study was different in that it followed incident patients from time of transplant, with measurements at baseline and one year, adding further information about what happens to PWV over time. Our results indicate that changes in aortic stiffness may be slowed with successful transplantation, consistent with two previous smaller studies over a shorter time period of 3 months [20, 21]. In the study by Ignace, et al. aortic stiffness changes post transplantation were dependent on age, with improvement seen only in transplant recipients older than 50 years [21]. A previous study by Zoungas, et al. observed aortic PWV in patients over 1 year showing a slight improvement, but the majority of these patients were on cyclosporine [15]. Our study has similar findings but in tacrolimus treated patients, which is important since tacrolimus is the most commonly prescribed calcineurin inhibitor, used as the initial choice in 91 % of patients in the United States [1]. To our knowledge this is the first study to report carotid PWV in a cohort entirely maintained on tacrolimus of this duration. A Japanese study of 58 patients on tacrolimus followed only up to 6 months post transplantation showed a decrease in brachial-ankle PWV in a low risk population of younger (mean age 40.5 years) kidney transplant recipients [22].

In contrast to our study, a study in Poland found that carotid PWV increased with time post transplantation, but these patients were already a median time of 36 months post transplantation at time of enrollment, differing from our population of newly transplanted patients. Similar PWV values to those in our study were observed in the 61 kidney transplant recipients with an eGFR of 55 mL/min/1.73 m^2^, where the initial measured carotid PWV was 9.1 m/s. In this group, however, PWV increased to 9.8 m/s when measured 24 months later [23]. One possible explanation for this finding was the prolonged exposure to transplant immunosuppression. Indeed, some studies have examined the effects of different classes of immunosuppression on aortic stiffness. In a randomized study in France, low cardiovascular risk groups of newly transplanted kidney patients were maintained on cyclosporine or randomized to sirolimus at week 12 and followed for a variety of cardiovascular related outcomes, including carotid PWV, for 1 year. Those in the sirolimus group had significantly lower PWV at follow up [24]. Similarly, another study compared carotid PWV in kidney transplant recipients on belatacept versus calcineurin inhibitors, and found after 5 years post transplantation, more patients in the calcineurin inhibitor group had a PWV greater than 8.1 m/s than in the belatacept group [25]. These studies support that calcineurin inhibitors may have more detrimental cardiovascular effects long term.

Strengths of our study included the prospective longitudinal design and duration of follow up, including the fact that all patients started follow up at time of their transplant, so they all had been exposed to transplant immunosuppression the same length of time. In addition, a large percent of the cohort was black, allowing study of a population not frequently available in other cohorts of kidney transplant recipients. A weakness was our inability to adjust for multiple covariates without overfitting the model due to our relatively small sample size, though we were able to adjust for all demographics and important cardiovascular risk factors like diabetes and pre-existing cardiovascular disease. Also due to the small number of cardiovascular events in this study current cohort, we were not able to test associations of PWV with these. In addition, overall our population was a low risk population for acute rejection and low risk for immediate cardiovascular events due to our selection process pre transplant.

## Conclusions

In a prospective study of new kidney transplant recipients treated with tacrolimus, we showed that there was no change in aortic PWV over the first year post kidney transplantation, suggesting arterial stiffness does not progress during this time period in a cohort of low risk individuals for cardio-renal outcomes. Further prospective research regarding arterial stiffness after transplant would be helpful to better understand the impact of transplant on cardiovascular risk. Specifically studies with longer follow-up time and with adequate number of cardiovascular events are necessary. Ultimately this may help identify kidney transplant patients at higher risk for CVD events and to target for intervention.

## Abbreviations

CKD: Chronic kidney disease
CVD: Cardiovascular disease
ESRD: End stage renal disease
PWV: Pulse wave velocity
VUMC: Vanderbilt University Medical Center
eGFR: Estimated glomerular filtration rate
